# Computational assembly of a human Cytomegalovirus vaccine upon experimental epitope legacy

**DOI:** 10.1186/s12859-019-3052-6

**Published:** 2019-12-10

**Authors:** Monica J. Quinzo, Esther M. Lafuente, Pilar Zuluaga, Darren R. Flower, Pedro A. Reche

**Affiliations:** 1 Faculty of Medicine, University Complutense of Madrid, Pza Ramon y Cajal, s/n, 28040 Madrid, Spain; 2 School of Life and Health Sciences, Aston University, Aston Triangle, Birmingham, B4 7ET UK

**Keywords:** HCMV, Epitopes, Vaccine, Prediction

## Abstract

**Background:**

Human Cytomegalovirus (HCMV) is a ubiquitous herpesvirus affecting approximately 90% of the world population. HCMV causes disease in immunologically naive and immunosuppressed patients. The prevention, diagnosis and therapy of HCMV infection are thus crucial to public health. The availability of effective prophylactic and therapeutic treatments remain a significant challenge and no vaccine is currently available. Here, we sought to define an epitope-based vaccine against HCMV, eliciting B and T cell responses, from experimentally defined HCMV-specific epitopes.

**Results:**

We selected 398 and 790 experimentally validated HCMV-specific B and T cell epitopes, respectively, from available epitope resources and apply a knowledge-based approach in combination with immunoinformatic predictions to ensemble a universal vaccine against HCMV. The T cell component consists of 6 CD8 and 6 CD4 T cell epitopes that are conserved among HCMV strains. All CD8 T cell epitopes were reported to induce cytotoxic activity, are derived from early expressed genes and are predicted to provide population protection coverage over 97%. The CD4 T cell epitopes are derived from HCMV structural proteins and provide a population protection coverage over 92%. The B cell component consists of just 3 B cell epitopes from the ectodomain of glycoproteins L and H that are highly flexible and exposed to the solvent.

**Conclusions:**

We have defined a multiantigenic epitope vaccine ensemble against the HCMV that should elicit T and B cell responses in the entire population. Importantly, although we arrived to this epitope ensemble with the help of computational predictions, the actual epitopes are not predicted but are known to be immunogenic.

## Background

Human Cytomegalovirus (HCMV) seroprevalence is 50–90% in the adult population. HCMV can be transmitted via saliva, sexual contact, placental transfer, breastfeeding, blood transfusion, solid-organ transplantation or hematopoietic stem cell transplantation. The main risk factors for HCMV infection, reactivation and disease are: immune-naive state, immunosuppressive regimens, organ transplants and co-infection [1]. The prevalence of congenital HCMV infection has been estimated between 0.5–0.7% in the US, Canada and Western Europe and between 1 and 2% in South America, Africa and Asia. Around 13% of infected infants are symptomatic with a wide range of phenotypes, including prematurity, intrauterine growth retardation, hepatomegaly, splenomegaly, thrombocytopenia, microcephaly, chorioretinitis, sensorineural hearing loss and focal neurologic deficits [2].

HCMV, or human herpesvirus 5, is a beta herpesvirus consisting of a 235 Kpb double-stranded linear DNA core. HCMV genome is among the longest and most complex genomes of all human viruses, due to the diversity of wild-type strains in intrahost and interhost HCMV populations. The HCMV genome is translated in 3 overlapping phases (IE-immediate early: 0-2 h; E-early: < 24 h; L-late: > 24 h) giving rise to RNAs and proteins with a structural and/or a functional role in different stages of the viral cycle [3]. Davidson et al. [4] estimate that the wild-type HCMV genome carries 164–167 coding mRNAs accounting for one third of transcription, while 4 large non-coding RNAs account for 65.1%.

Although HCMV can reside in both, myeloid and lymphoid lineages, monocytes are its primary target. HCMV reactivation and dissemination may occur after infected monocytes migrate into tissues and differentiate into macrophages since, unlike monocytes, they are permissive for viral gene expression [5]. Initial viral tethering occurs by engagement of glycoprotein M/N to heparin proteoglycans, followed by binding of monocyte β1 and β2 integrins and epidermal growth factor receptor (EGFR). This binding activates downstream receptor signalling, which prompts viral entry and increases cellular motility, thus facilitating viral dissemination [6]. Once primary infection begins, there is a rapid innate response. Toll-like receptors (TLRs) interact with viral DNA starting the production of inflammatory cytokines, such as type I interferons (IFNs), which leads to an antiviral state and activates dendritic cells (DCs), macrophages and natural killer (NK) cells [7].

HCMV-specific adaptive immunity is required for long-lasting protective immunological memory, which prevents from reinfection, reactivation, uncontrolled replication and serious disease. Protection against HCMV is correlated with high frequencies of CD8 cytotoxic T lymphocytes (CTLs) specific for immediate-early 1 protein (IE-1) and 65 KDa phosphoprotein (pp65) as well as type 1 CD4 T helper (Th1) cells specific for glycoprotein B (gB), TLR14 and UL16, which also exhibit cytotoxic activity [8–11]. Unlike T cells, B cells recognize solvent-exposed epitopes in target antigens. This recognition promotes B cell activation resulting in the secretion of antibodies (Abs) with the same specificity. Some protective anti-HCMV Abs have been shown to recognize envelope glycoprotein B (gB) and glycoprotein H (gH) [12].

Despite eliciting strong immune responses, HCMV has a large evasion armoury that is responsible for the resilience of the virus and its prevalence in the population. HCMV interferes with cytokine pathways, NK cell activation and antigen processing and presentation [13]. In addition, several studies point that numerous cycles of HCMV reactivation can lead to an early state of immune senescence, characterized by the decline of immune responsiveness, as well as the reduction in the levels of naive cells. This feature could be behind the association between chronic subclinical infection and long-term diseases such as atherosclerosis, chronic graft rejection, autoimmunity and certain neoplasias [14, 15].

Despite much effort, an effective treatment for HCMV disease remains a significant challenge. The most effective approach to prevent infection, transmission or reactivation in immune-naive or immunosuppressed individuals will be a multifunctional HCMV vaccine [16]. Currently, such a vaccine is not available. Vaccine development requires much effort, resource, and knowledge; yet the process can be facilitated greatly using immunoinformatics and related computational approaches [17–19]. Such approaches are particularly relevant for the design of epitope-based vaccines, which stand out for their safety and selectivity [20, 21]. The design of epitope ensemble vaccines relies on sophisticated immunoinformatics tools, often based on machine learning, able to identify the majority of potential T and B cell epitopes from pathogen genomes [22, 23]. However, such predictions still require experimental validation, with only a few potential epitopes actually being immunogenic, and thus suitable for vaccine design [24].

Here, we designed multi-functional epitope-based vaccine for HCMV through an approach that combines legacy experimentation with immunoinformatic predictions [25–31]. The approach uses previously validated epitopes of proven immunogenicity obtained from public databases. A long list of experimentally-determined T-cell and B-cell epitopes is successively pruned by applying a series of sequence conservation, structural and immunological criteria. Subsequently, highly conserved epitopes meeting the required criteria are combined to minimise epitope number while retaining 90% or greater population protection coverage [25–31]. Our putative epitope ensemble vaccine should prove a viable starting point for the development of an effective vaccine against HCMV.

## Results

### HCMV amino acid sequence variability

Compared to other organisms, viruses have a high replication rate, displaying great sequence variability. This feature facilitates immune evasion and can hinder the development of vaccines providing protection to all strains. Such immune evasion can be better countered back with vaccines consisting of non-variable epitopes [20]. We analysed the amino acid sequence variability of HCMV proteins as a way of identifying non-variable epitopes (details in Methods). Briefly, we first clustered all HCMV protein sequences (50,623) around a reference HCMV genome (NC_006273), obtaining representative protein clusters (162) for all but 9 of the ORFs included in the selected reference HCMV genome. We then produced multiple sequence alignments (MSAs) and subjected them to sequence variability analysis. We found that only 601 out of 62,196 residues had a variability H ≥ 0.5 (a site with H ≤ 0.5 is considered to be conserved). This extremely low variability is unexpected, even for a dsDNA virus, facilitating the selection of conserved epitopes for vaccine design. After these analyses, we selected only those epitopes that did not have any single residue with H ≥ 0.5.

### Selection of CD8 T cell epitopes

We retrieved from IEDB (https://www.iedb.org/) 20 experimentally verified HCMV-specific CD8 T cell epitopes from 499 available epitopes after the following search criteria: A) recognition by human subjects exposed to the virus and B) induction of epitope specific CD8 T cells with killing activity over cells infected with HCMV. This type of selection guaranties that CD8 T cell epitopes are appropriately processed and presented by both, dendritic cells priming epitope-specific CD8 T cells and infected target cells. Of those, we discarded any peptide with variable residues and size out of the 9–11 residue-range as they are unlikely to bind class I human leukocytes antigen (HLA I) molecules. Thus, we retained 9 conserved CD8 T cell epitopes with a size between 9 and 11 residues that were subjected to HLA I binding predictions and population protection coverage (PPC), analyses (details in Methods). We found that just a single epitope (QYDPVAALF) could reach a PPC that is at the least of 66.71% (Table 1). We computed PPCs for 5 distinct ethnic groups in the USA populations and thus the minimum PPC is that reached in the group with the lowest coverage (details in Methods). The combined minimum PPC of all the peptides is 92.99% while the PPC for each ethnic group is: 99.76% for Blacks, 96.16% for Caucasians, 98.18% for Hispanics, 92.99% for Native North Americans and 99.96 for Asians. The average PPC for the USA population is 97.41% and it can be reached by the combination of 6 epitopes: QYDPVAALF, NLVPMVATV, TTVYPPSSTAK, HERNGFTVL, QTVTSTPVQGR, TPRVTGGGAM.

**Table 1:** HLA I binding profiles of conserved and experimentally verified HCMV-specific CTL epitopes

Epitope	Antigen gene	Antigen accession	^a^ HLA I restriction	^b^ Extended HLA I restriction	^c^ PPC (%)
QYDPVAALF	UL83	Q6SW59	A*24:02, A*01:01, C*04:01	A*:01:01, A*24:02, B*15:16, B*38:01, B*39011, B*3909, B*5801, C*04:01, C*07:02	66.71
NLVPMVATV	UL83	Q6SW59	A*02:01, A*02:02, A*02:03, A*02:06, A*02:11, A*02:12, A*02:16, A*02:19, A*24:02, A*69:01, A*03, B*07:02	A*02:01, A*02:02, A*02:03, A*02:05, A*02:06, A*02:09, A*02:14, A*68:02, A*02:12, A*02:16, A*02:19, A*02:11, A*69:01, A*24:02, B*07:02	58.98
TTVYPPSSTAK	UL32	Q6SW99	A*03:01	A*0301, B*15:02, B*15:08, C*07:02	34.47
HERNGFTVL	UL83	Q6SW59	B*40:01, B*40:02, B*60	A*2902, B*0702, B*1510, B*40:01, B*40:02	24.21
TPRVTGGGAM	UL83	Q6SW59	A*02:01, B*07:02	A*02:01, B*07:02, B*55:02, B*27:06	9.79
CEDVPSGKL	UL83	Q6SW59	B*40:01, A*03, B*60	B*38:01, B*40:01	2.78
QTVTSTPVQGR	UL32	Q6SW99	A*68:01	A*33:01, A*68:01	1.12
AELEGVWQPA	UL83	Q6SW59	B*40:06	A*02:09, B*40:06	0

^a^Experimental restriction found in IEDB; ^b^ Experimental plus predicted HLA I restriction/binding (details in Methods); ^c^ PPC was computed independently for 5 ethnic groups in the USA population using EPISOPT [27] and here we report the lowest PPC value

### Selection of CD4 T cell epitopes

We obtained from IEDB (https://www.iedb.org/) 291 experimentally validated HCMV-specific CD4 T cell epitopes recognized by humans exposed to the HCMV. Of those, we selected 91 epitopes belonging to structural proteins for size and conservation analysis. Thus, we identified 77 conserved epitopes with a size between 9 and 21 amino acids, the usual length of peptides restricted by class II HLA (HLA II) molecules. These 77 epitopes belonged to pp65 (UL83) and gB (UL55). No conserved epitopes were identified in other structural proteins. Although these 77 epitope peptides were unique, some were largely overlapping. Therefore, we applied a clustering-based procedure (details in Methods) to identify shared epitopes defined by overlapping peptides. Thus, we proceeded with 37 CD4 T cell epitopes, 15 derived upon clusters, for HLA II binding and PPC analyses. In Table 2 we only report epitopes with PPC ≥ 10%. The maximum PPC obtained with all peptides was 92.49%. However, we found that only 6 epitopes from the 65 KDa phosphoprotein were necessary to achieve the same PPC: SIYVYALPLKMLNIP, KLFMHVTLGSDVEEDLTMTR, YQEFFWDANDIYRIF, LPLKMLNIPSINVHH, CSMENTRATKMQVIG and AGILARNLVPMVATV.

**Table 2:** Predicted HLA II binding profile of conserved and experimentally verified HCMV-specific CD4 T cell epitopes

Epitope	Antigen gene	Antigen accession	^a^ HLA II restriction	^b^ Extended HLA II restriction	^c^ PPC (%)
SIYVYALPLKMLNIP	UL83	Q6SW59	DRB1*07, DRB1*15:01	DRB1*01:01, DRB1*03:01, DRB1*07:01, DRB1*11:01, DRB1*15:01	63.95
KLFMHVTLGSDVEEDLTMTR	UL83	Q6SW59	DRB1*03	DRB1*03:01, DRB1*04:05, DQA1*05:01/ DQB1*02:01, DQA1*05:01/ DQB1*03:01	48.03
LPVADAVIHASGKQMWQARL	UL83	Q6SW59	DRB1*03	DRB1*01:01, DQA1*05:01/ DQB1*02:01, DQA1*05:01/ DQB1*03:01	42.11
GPISGHVLKAVFSRG	UL83	Q6SW59	HLA class II	DRB1*01:01, DQA1*05:01/ DQB1*02:01, DQA1*05:01/ DQB1*03:01	42.11
CSMENTRATKMQVIG	UL83	Q6SW59	HLA class II	DRB1*07:01, DQA1*01:02/ DQB1*06:02	37.99
FTSHEHFGLLCPKSI	UL83	Q6SW59	HLA class II	DRB1*01:01, DRB1*07:01, DRB1*09:01, DRB5*01:01	34.02
YQEFFWDANDIYRIF	UL83	Q6SW59	DRB1*01	DRB1*04:01, DRB3*01:01, DQA1*01:01/ DQB1*05:01	27.69
RNGFTVLCPKNMIIK	UL83	Q6SW59	HLA-DR	DRB1*01:01, DRB1*09:01, DRB1*11:01	27.09
RLLQTGIHVRVSQPS	UL83	Q6SW59	DRB1*15, DQA1*01:02/ DQB1*06:02	DQA1*01:02/ DQB1*06:02	24.17
LRQYDPVAALFFFDI	UL83	Q6SW59	DRB1*07	DRB1*07:01, DRB1*09:01,	24.01
LPLKMLNIPSINVHH	UL83	Q6SW59	DRB1*03	DRB1*01:01, DRB1*04:04, DRB1*09:01	21.87
QNLKYQEFFWDANDI	UL83	Q6SW59	HLA class II	DRB5*01:01, DQA1*01:01/ DQB1*05:01	18.56
EPDVYYTSAFVFPTK	UL83	Q6SW59	DRB1*07	DRB1*15:01	18.41
MLDVAFTSHEHFGLL	UL83	Q6SW59	HLA class II	DRB1*07:01	18.23
SDVEEDLTMTRNPQP	UL83	Q6SW59	DRB1*03	DRB1*03:01	17.84
AGILARNLVPMVATV	UL83	Q6SW59	DRB1*11, DRB3*02:02	DRB1*01:01, DRB3*02:02	11.53
EHPTFTSQYRIQGKL	UL83	Q6SW59	DRB1*11:01	DRB1*11:01	10.54
TSQYRIQGKLEYRHT	UL83	Q6SW59	DRB1*04, DRB1*13	DRB1*11:01	10.54

^a^ Experimental restriction found in IEDB; ^b^ Experimental plus predicted HLA II restriction/binding (details in Methods); ^c^ PPC was computed for 21 different ethnicities around the world

### Selection of B cell epitopes

We found 398 experimentally validated HCMV-specific unique linear B cell epitopes generated during a natural infection. Of those, we focused on conserved epitopes mapping onto the ectodomain of envelope antigens so that they could induce protective Abs recognizing viral particles. Thus, we found 99 epitopes located in the ectodomains of glycoprotein H (UL75), glycoprotein L (UL115), glycoprotein B (UL55), glycoprotein M (UL100), glycoprotein UL4 (UL4), glycoprotein UL1 (UL1), TLR10 (IRL10) and TRL12 (IRL12). We clustered these epitopes to identify common overlapping epitopes, finding only two epitopes from 2 sets of 4 and 7 overlapping epitopes (see Methods). All remaining 90 epitopes were fragmented into 9mers overlapping 8 amino acids, sought for conservation and clustered to identify the longest conserved fragment. Thus, we identified 15 conserved epitopes for which we computed their flexibility and accessibility (Table 3).

**Table 3:** Conserved and experimentally verified B cell epitopes from HCMV envelope proteins

Epitope	Antigen gene	^a^ Accession number	^b^ PDB	^c^ Flexibility	^d^ Accessibility (%)
VSIDDDTPML	UL75	Q6SW67	5VOB: A [238–247]	0.131	25.37
TNQYLIKGISYPVST	UL75	Q6SW67	5VOB: A [592–606]	0.09	32.63
AFHLLLNTYGR	UL75	Q6SW67	5VOB: A [37–47]	1.618	55.08
IFTEHVLGFELVPPS	UL115	F5HCH8	5VOB: B [173–187]	−0.275	11.71
TANQNPSPPWSKLTYSKPH	UL130	F5HCP3	5VOB: D [32–50]	0.104	39.64
WSTLTANQNPSPPWSKLTY	UL130	F5HCP3	5VOB: D [28–46]	0.138	33.37
FTYDTLRGYINRALA	UL55	F5HB53	5C6T: A [486–500]	− 0.644	64.02
LRGYINRALAQIAEA	UL55	F5HB53	5C6T: A [491–505]	−0.645	68.14
NRALAQIAEAWCVDQ	UL55	F5HB53	5C6T: A [496–510]	−0.724	63.41
QIAEAWCVDQRRTLE	UL55	F5HB53	5C6T: A [501–515]	−0.904	56.29
SAILSAIYNKPIAAR	UL55	F5HB53	5C6T: A [526–540]	−1.187	40.75
SKINPSAILSAIYNK	UL55	F5HB53	5C6T: A [521–535]	−1.241	46.13
VFKELSKINPSAILS	UL55	F5HB53	5C6T: A [516–530]	−1.322	38.57
YAQLQFTYDTLRGYI	UL55	F5HB53	5C6T: A [481–495]	−0.734	53.73
NVTFRGLQNKTEDFL	UL4	Q6SWC6	–	0.442	19.39

^a^ Accession number from UniProtKB database. ^b^ Tertiary structure of the antigen (PDB code) with epitope location in square brackets. ^c^ Average flexibility (*F*_*b*_, Eq. 3) of epitope in arbitrary units. ^d^ Average relative solvent-exposed accessibility of epitope in percentage (*A*_*b,*_ Eq. 4). The epitopes AFHLLLNTYGR and WSTLTANQNPSPPWSKLTY, were part of the epitopes AASEALDPHAFHLLLNTYGR and SWSTLTANQNPSPPWSKLTY, respectively. Accessibility and flexibility of NVTFRGLQNKTEDFL was predicted upon the antigen amino acid sequence as it did not map onto any 3D-structure (details in Methods)

Since only one epitope (AFHLLLNTYGR) had a flexibility ≥1.0 and an accessibility ≥48%, determining their location in highly flexible and solvent-exposed regions [25], we sought for potential B cell epitopes from available crystal structures of HCMV envelope proteins (details in Methods) predicting 2 B cell epitopes, one in the ectodomains of the gH and another one in the ectodomain of the gL, that were also conserved (Table 4).

**Table 4:** Predicted conserved B cell epitopes from HCMV envelope proteins

Epitope	Antigen gene	^a^ Accession number	^b^ PDB	^c^Flexibility	^d^Accessibility (%)
TYNSSLRNS	UL75	Q6SW67	5VOB: A [11–28]	1.752	48.58
TPEAANSVLLD	UL115	F5HCH8	5VOB: B [57–69]	1.397	58.36

^a^Accession number from UniProtKB database. ^b^ Tertiary structure of the antigen (PDB code) with epitope location in square brackets. ^c^ Average flexibility of epitope in arbitrary unit (*F*_*b*_, Eq. 3). ^d^ Average solvent-exposed accessibility of epitope in percentage (*A*_*b*_, Eq. 4)

## Discussion

There have been considerable efforts to develop a vaccine against HCMV, ranging from using attenuated viruses to various viral subunits [16]. However, there is currently no effective vaccine against HCMV. Subunit vaccines based on gB have shown 50% efficacy in preventing primary infection in young mothers and transplantation recipients, but they cannot prevent successive infections nor do they produce long-term protection [32, 33]. Live recombinant vaccines based on replication-deficient viral vectors (e.g. poxvirus, adenovirus) encoding multiple HCMV-specific epitopes have also been tested but they were poorly immunogenic and only after long periods of stimulation and expansion [34]. In this context, we designed a multi-functional epitope-based vaccine against the HCMV.

**Fig. 1:**
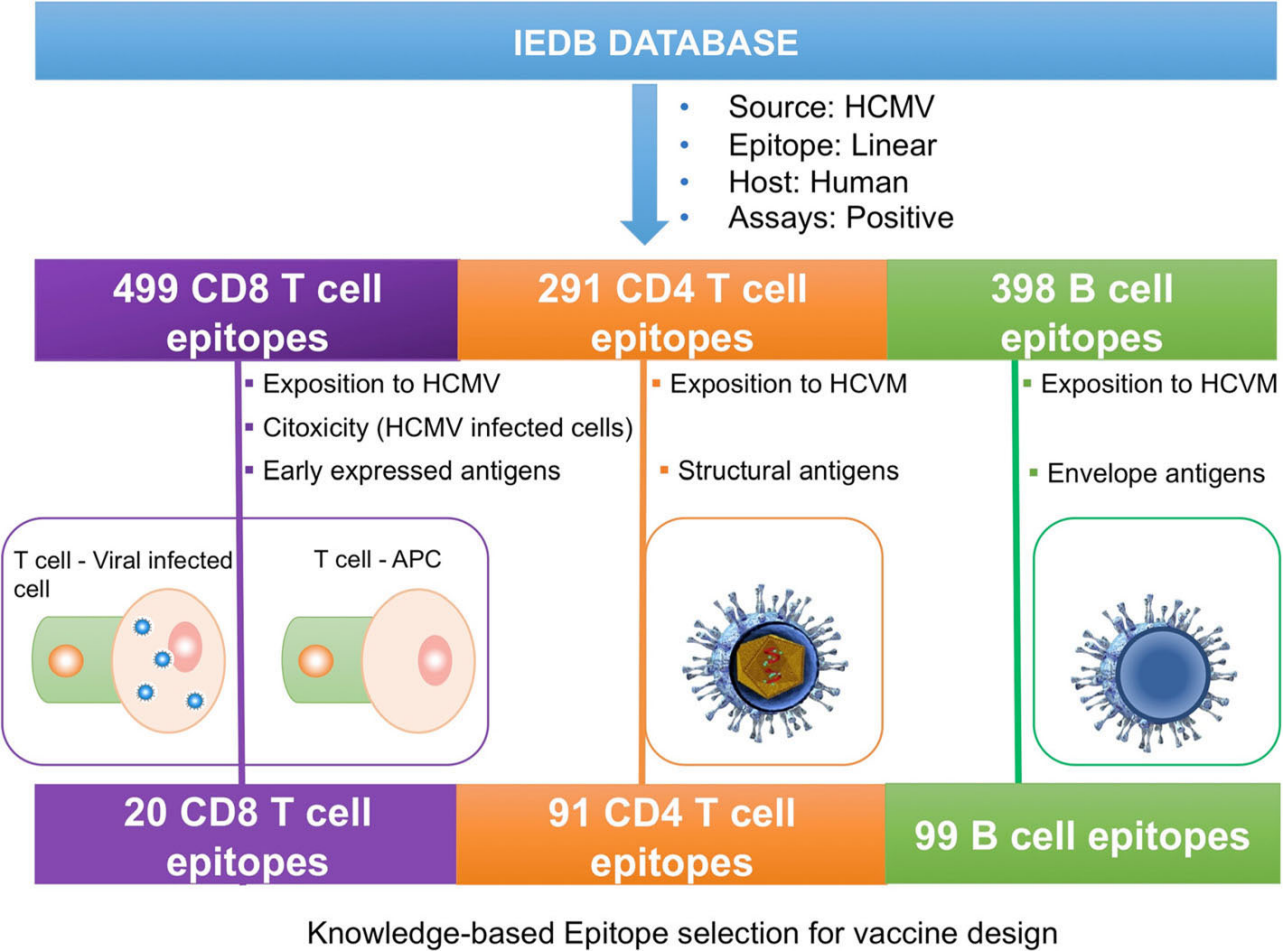
Mapping of predicted (purple and blue) and experimentally defined (red) B cell epitopes on the tertiary structure of the gH and gL as part of the pentameric complex UL75/UL115/UL128/UL130/UL131A. B cell epitopes are respresented as sticks over a background of ribbons

The main advantage of the epitope-based formulations is their exquisite selectivity as well as the possibility of inducing immune responses to subdominant epitopes and to various antigens at the same time. Moreover, they have been proposed to be safer than traditional vaccines [20, 35]. Developing epitope-based vaccines is bound to the need to identify pathogen-specific epitopes within the relevant antigens, which, in spite of the available epitope prediction methods, is only achieved after laborious and costly experiments [22]. CD8 T cell epitope prediction methods are widely regarded as the most accurate and yet only 10% of predicted T cell epitopes are found to be immunogenic [36]. To bypass this problem, we formulated an epitope vaccine ensemble for HCMV through a computer-assisted approach that feeds on previously identified epitopes readily available in specialized databases [37–40]. Clearly, the main advantage of this approach is the saving of time and resources as it depends on experimentally-validated epitopes. We first applied this approach for human immunodeficiency − 1 virus and hepatitis C virus, considering only CD8 T cell epitope vaccines [27, 29], later extending this to influenza A virus considering also CD4 T cell epitopes [31] and more recently to Epstein-Bar virus including B cell epitopes [25]. The keystone of this approach is to select conserved epitopes that are likely to induce protective immune responses (Fig. 1). In the specific case of HCMV, we selected CD8 T cell epitopes that are processed and presented both by antigen presenting cells (APCs) and HCMV infected cells, mediate cytotoxic activity and are derived from early expressed antigens. Consequently, memory CD8 T cells elicited by these epitopes will detect and kill infected cells early on avoiding virus dissemination. For CD4 T cell epitopes, we focused on epitopes presented by APCs from structural proteins so that they will provide early and effective help. Similarly, we only considered B cell epitopes mapping onto the ectodomain of envelope proteins so that they can elicit Abs recognizing the entire virus and block infection.

The epitopes obtained from the initial selection steps were subjected to different analysis for vaccine inclusion. The final epitope ensemble vaccine that we propose consists of 6 CD8 T cell epitopes, 6 CD4 T cell epitopes and 3 B cell epitopes (See Table 5). Conserved T cell epitopes were included in the ensemble for their ability to be presented by multiple HLA molecules providing maximum PPC. Thus, the CD4 and CD8 T cell epitope components are predicted to elicit responses in at least 90% of the population, regardless of their ethnicity. This level of response assumes that epitopes shown to be immunogenic in a specific HLA context will be also immunogenic in all the other HLA contexts defined by their HLA binding profile. Likewise, it assumes that antigen processing and appropriated epitope release remain the same in any HLA context. There is considerable evidence for these assumptions [19, 29]. However, since epitope-HLA binding profiles are predicted, they will need confirmation for further vaccine development.

**Table 5:** Epitope ensemble vaccine for HCMV

Epitope	Antigen gene	Antigen accession	BLAST hit human (%)	BLAST hit HMP (%)	PPC (%)
CD8 T cell epitope vaccine component					
QYDPVAALF	UL83	Q6SW59	NP_060360.3 (66.67)	ETS98202.1 (78.00)	66.71
NLVPMVATV	UL83	Q6SW59	AMD82163.1 (66.67)	KWZ77571.1 (78.00)	58.98
TTVYPPSSTAK	UL32	Q6SW99	CAC15059.1 (63.64)	OFS88048.1 (72.73)	34.47
HERNGFTVL	UL83	Q6SW59	XP_016879611.1 (77.78)	EGG81521.1 (77.78)	24.21
TPRVTGGGAM	UL83	Q6SW59	AGP01160.1 (70.00)	EJD64485.1 (80.00)	9.79
QTVTSTPVQGR	UL32	Q6SW99	NP_005892.1 (63.64)	EHY57399.1 (82.00)	1.12
CD4 T cell epitope vaccine component					
SIYVYALPLKMLNIP	UL83	Q6SW59	XP_006724177.2 (46.67)	AEA20835.1 (73.33)	63.95
KLFMHVTLGSDVEEDLTMTR	UL83	Q6SW59	EAX02867.1 (45.00)	ERJ00718.1 (45.00)	48.03
YQEFFWDANDIYRIF	UL83	Q6SW59	EAW98902.1 (46.67)	EIJ68880.1 (60.00)	27.69
LPLKMLNIPSINVHH	UL83	Q6SW59	EAX02055.1 (46.67)	EEU31012.1 (73.33)	21.87
CSMENTRATKMQVIG	UL83	Q6SW59	EAW54200.1 (53.33)	EGL44246.1 (73.33)	37.99
AGILARNLVPMVATV	UL83	Q6SW59	BAC11142.1 (46.67)	EFV13316.1 (73.33)	11.53
B cell epitope vaccine component					
AFHLLLNTYGR	UL75	Q6SW67	EAW77317.1 (63.64)	KXA44279.1 (63.64)	
*TYNSSLRNS	UL75	Q6SW67	AHA56189.1 (78.00)	EFE23794.1 (90.00)	
*TPEAANSVLLD	UL115	F5HCH8	NP_001017403.1 (72.73)	OFU98142.1 (72.73)	

We show closest blast hits to human Proteins and Human Microbiome Proteins. The B cell epitope component contains one epitope validated experimentally and 2 predicted epitopes (*). PPC of the entire CD8 and CD4 T cell component is 97.41 and 92.49%

Conserved B cell epitopes in epitope ensemble vaccine were selected after flexibility and accessibility criteria and included one experimental epitope on gH and 2 predicted epitopes, one on gH and another on gL (Table 5). The criteria of flexibility and accessibility that we applied were optimized to identify unstructured B cell epitopes lying in flexible and solvent exposed loop regions of the corresponding native antigens [25]. Consequently, these B-epitopes can be used as immunogens isolated from the antigen, e.g. as peptides, to induce the production of Abs that are likely cross-reactive with the native antigen [22].

All the epitopes in the proposed epitope ensemble are highly conserved to avoid or reduce immune evasion caused by viral genetic drift. Interestingly, we found that despite HCMV having very low sequence variability (1% of variable residues) only 40% of the selected T cell epitopes and 15% of the selected B cell epitopes are conserved. These results indicate that sequence variability enables HCMV to escape the immune response, particularly the Ab response. They also highlight the crucial role of T cell responses in the control of HCMV in infected individuals.

Our epitope ensemble vaccine is multiantigenic, targeting 4 different HCMV proteins: pp65 (UL83), 150KDa phosphoprotein (pp150, UL32), envelope gL (UL115) and envelope gH (UL75). There are 2 antigens represented in the CD8 T cell epitope component (pp65 and pp150) and 2 antigens in B cell epitope component (gL and gH). However, CD4 T cell component only contains epitopes from the pp65. Arguably, it would have been better to include epitopes from some other antigens in the CD4 T cell component. However, the selected CD4 T cell epitopes do provide the maximum PPC and ought to offer effective help to both CD8 T cells and B cells.

**Fig. 2:**
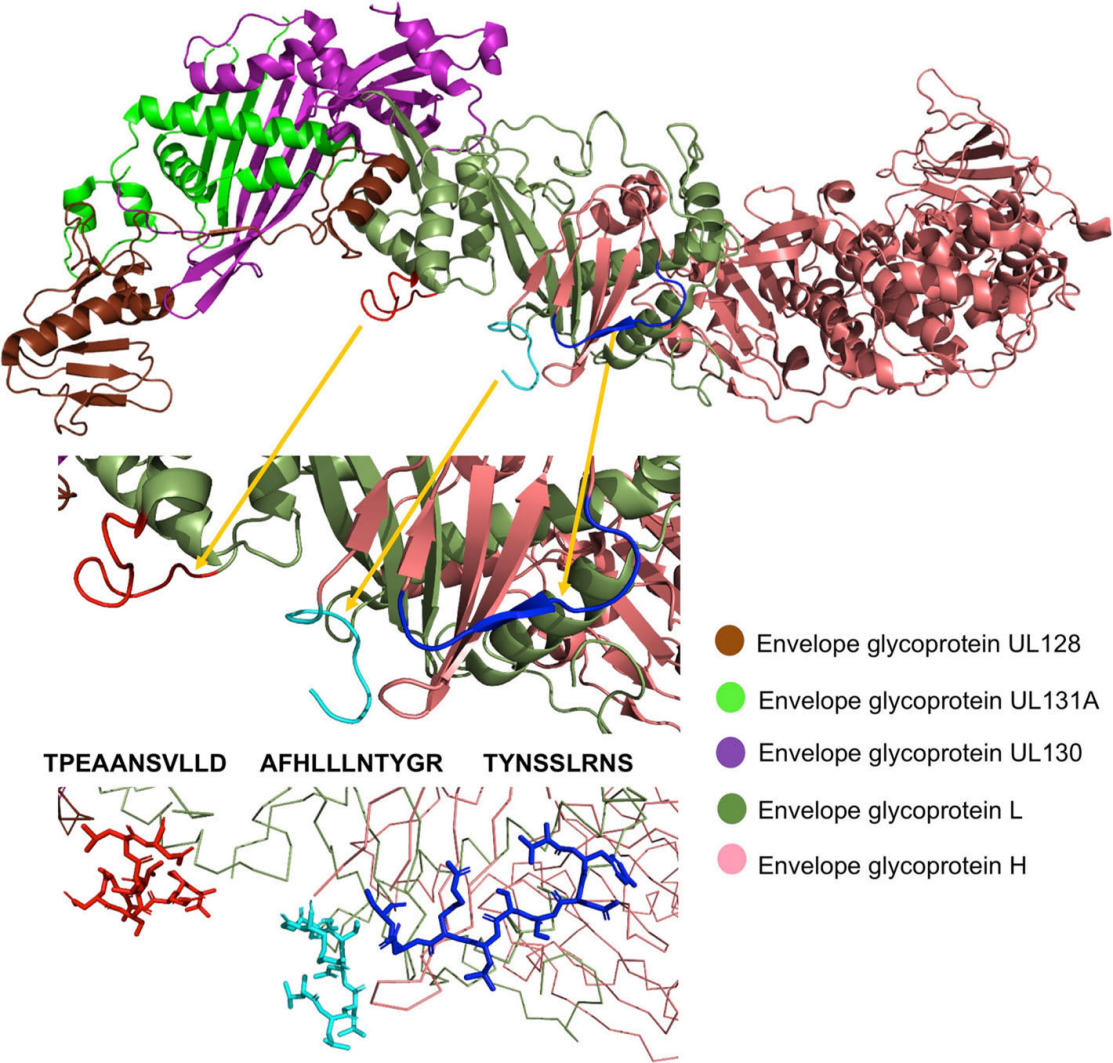
Knowledge-based selection of experimental epitopes for HCMV vaccine design. Experimental epitopes were obtained form IEDB and selected to identify those that are more likely to induce protective immunity in humans. CD8 T cell epitopes were identified upon searches that guarantee that were processed and presented early by APCs (immunogen exposition) and by target cells (mediate cytotoxic activity of cells infected with HCMV). CD4 T cell epitopes were selected for being recognized by HCMV exposed subjects and belonging to structural proteins, so that they will provide early effective help. B cell epitopes were also selected for being recognized by HCMV exposed subjects and mapping onto the ectodomain of envelope proteins so that they can induce neutralizing antibodies

Three of the targeted antigens (UL83, UL115 and UL75) have been included in other vaccines currently undergoing clinical trials, highlighting the importance of these antigens as components of a HCMV-specific vaccine. The viral protein pp65 (UL83) is delivered to infected cells as a virion component and rapidly moves to the nucleus where it antagonizes the cellular antiviral response through the NF-κB pathway [41]. The viral protein pp150 (UL32) associates with the nuclear viral capsids before DNA encapsidation and later protects nucleocapsids along secondary envelopment at the assembly compartment [42]. gH and gL are part of the gH/gL/gO trimeric complex and the gH/gL/UL128/UL130/UL131A pentameric complex which are important for viral entry into fibroblasts (trimeric complex) and epithelial and endothelial cells (pentameric complex) [43]. It has been shown that antibodies targeting gL/gH can hinder assembly of both complexes blocking HCMV entry into host cells [43]. Interestingly, the three B cell epitopes selected in this study are in regions of gL and gH interacting with proteins of the trimeric and pentameric complexes (Fig. 2). Thereby, we speculate that Abs elicited by these 3 B cell epitopes will block HCMV entry in fibroblasts and epithelial and endothelial cells. HCMV has additional proteins that are also important for entry in other cell types such as gB and the gM/gN complex that are involved in HCMV infection of monocytes [43]. It would have been desirable to have these HCMV envelope proteins represented in the B cell epitope component of our vaccine. Unfortunately, we could not identify conserved B cell epitopes meeting our criteria of flexibility and accessibility in such proteins.

A potential adverse effect of vaccines is that of inducing immune responses cross-reactive with self-antigens. Thereby, we verified that none of the included epitopes matched exactly human proteins or human microbiome proteins. The sequence similarity of all epitopes with human proteins is less than 80%; only two epitopes have a similarity over 80% with microbiome proteins. Since immune recognition is exquisitely specific, it can be disrupted by single amino acid mutation [44], and it is unlikely that the epitope ensemble proposed here will elicit harmful self-immune responses.

## Conclusions

We have assembled a HCMV vaccine consisting of 6 CD8 T, 6 CD4 T and 3 B cell epitopes from 4 different HCMV antigens. The epitopes do not match self proteins, are conserved and all but 2 B cell epitopes are experimentally verified and reported to be recognized by humans exposed to HCMV. This epitope ensemble was built using a knowledge-based, computer assisted approach aimed at identifying epitopes that are likely to induce protective adaptive immune responses. Thus, the T cell epitopes are predicted to provide a PPC over 90% and include CD8 T cell epitopes mediating cytoxicity against HCMV infected cells. The B cell epitopes are all in highly flexible and accessible regions of the ectodomain of gH and gL proteins which makes them suitable for inducing Abs cross-reactive with the relevant native antigens. Moreover, they are proximally located to regions involved in the assembly of key complexes for viral entry. Thus, Abs induced by these epitopes could be neutralizing and block infection.

We have sought to identify optimal epitope components for making a protective HCMV vaccine, but there remains a long road ahead prior to deploying a preventive vaccine. Epitope peptides are known to be poorly immunogenic and the epitope ensemble will have to be contained within a formulation capable of inducing potent innate and adaptive immune responses. An attractive formulation will be to encapsulate the T cell epitopes along with appropriated adjuvant on liposome-based nanoparticles, displaying the B cell epitopes on the outer surface [45].

## Methods

### Collection of HCMV-specific immunogenic epitopes and 3D-structures of HCMV envelope proteins

Experimentally confirmed HCMV-specific epitopes were obtained from IEDB [46]. We only considered epitopes producing positive assays with humans as the host. In addition, we applied different search criteria to B and T cell epitopes. For B cell epitopes, we considered any linear peptide from HCMV while we only considered HCMV-specific T cell epitopes that were elicited in humans exposed to the HCMV. In addition, for CD8 T cell epitopes, we restricted the selection to those that were reported to test positive on ^51^Cr cytotoxic assays with cells infected with HCMV (relation between epitope and antigen is source organism).

### Multiple sequence alignment of HCMV proteins and generation of consensus proteins through sequence variability analysis

We used CD-HIT [47] to cluster HCMV protein sequences (50,623) – obtained from NCBI taxonomy database (TAX ID: 10359) [48] and including the open reading frames (ORFs) of a reference HCMV genome (NC_006273)–, using an identity threshold of 85%. Subsequently, we selected those clusters containing reference sequences and produced multiple sequence alignments (MSA) using MUSCLE [49].

Sequence variability of the MSA was analysed per site/position using the Shannon Entropy (*H)* [50], as the variability metric (Eq. 1).

1 $$ H=-{\sum}_i^M{P}_i{Log}_2\left({P}_i\right) $$

where *P*_*i*_ is the fraction of residues of amino acid type *i* and M is the number of amino acid types. *H* ranges from 0 (only one amino acid type is present at that position) to 4.322 (every amino acid is equally represented in that position). Following these calculations, we masked in the reference HCMV proteome (NC_006273) any site with *H* ≥ 0.5, thus generating consensus sequences. HCMV epitopes that matched entirely with the consensus HCMV sequences were retained for subsequent analysis.

### Simplification of epitope datasets containing overlapping peptides

We used CD-HIT [47] to identify clusters of overlapping peptide sequences in the CD4 and B cell epitope datasets. MSAs generated after the relevant clusters were processed so that overlapping epitopes were then represented by the common core defined by the MSA. For CD4 T cell epitopes, the common core was extended up to a length 15 residues when needed, adding relevant N- and/or C-terminal residues. No common core longer than 15 residues was identified for overlapping CD4 T cell epitopes.

### Prediction of peptide HLA binding profiles and computation of population protection coverage

We predicted binding of CD8 T cell epitopes to 55 HLA I molecules using EPISOPT (http://imed.med.ucm.es/Tools/episopt.html) [27]. EPISOPT uses profile-motifs to predict peptide-MHC binding [51, 52] and considers peptides as HLA binders when their score is within the top 2% percentile. HLA I allele specific profile-motifs in EPISOPT only predict binding of 9mer peptides, which is the most common size of peptides found to bind HLA I molecules [53]. For longer peptides, HLA I binding profiles were obtained evaluating the binding of all 9mer peptides within the longer peptide. For CD4 T cell epitopes, we predicted peptide binding to a reference set of 27 HLA II molecules [54] with IEDB tools (http://tools.iedb.org/mhcii/). The reference set includes HLA II molecules belonging to HLA-DP, HLA-DQ and HLA-DR genes and a 5% percentile rank was used to assess binding. As the prediction method, we selected “IEDB recommended”. This method provides a consensus prediction which combines matrix and neural network-based models, when the relevant predictors are available, otherwise returning predictions provided by NetMHCIIpan [55]. For peptides longer than 15 residues, predicted HLA-II binding profiles corresponded to all 15-mers overlapping 14 amino acids contained in the longer peptide. Epitope population protection coverage (PPC) was computed with EPISOPT [27] for CD8 T cell epitopes and with the IEDB PPC tool for CD4 T cell epitopes (http://tools.iedb.org/tools/population/iedb_input) [56]. EPISOPT computes the PPC for 5 distinct ethnic groups prevalent in North America (Black, Caucasian, Hispanic, Asian and Native North American), accounting for linkage disequilibrium between HLA I alleles [27], and identifies epitope ensembles reaching a determined PPC. The IEDB PPC tool does not consider linkage disequilibrium between HLA II alleles but does include allele frequency for 21 different ethnicities around the world [56].

### Computation of flexibility and accessibility of B cell epitopes

The flexibility and accessibility of B cell epitopes was predicted using the relevant Protein Data Bank (PDB) files, when available, as described elsewhere [25]. Briefly, we computed normalized C*α* B-factors, *Z*_*Bi*_ (Eq. 2), after the PDBs and used them as a measure of flexibility:

2 $$ {Z}_{Bi}=\frac{\left({B}_i-{\mu}_B\right)}{\partial_B} $$

In Eq. 2, B_i_ is the B factor of the C*α* from residue *i*, obtained from relevant PDB, μ_B_ is the mean of C*α* B factors, and *∂*_*B*_ is the corresponding standard deviation. Likewise, we used NACCESS [57] to compute residue relative solvent accessibility (RSA) from the relevant PDBs.

Subsequently, we used Eq. 3 and 4 to compute an average flexibility (*F*_*b*_) and accessibility (*A*_*b*_), respectively, for each B cell epitope.

3 $$ {F}_b=\frac{\sum_{i=1}^{i=n}{Z}_{Bi}}{n} $$

4 $$ {A}_b=\frac{\sum_{i=1}^{i=n}{RSA}_i}{n} $$

where n is the total number of residues encompassed by the B cell epitope.

For B cell epitope sequences in antigens without solved tertiary structure, we predicted residue RSA and normalized B values with NetSurfP [58] and profBval [59], respectively, using as input the entire antigen sequence. Subsequently, we computed *F*_b_ and *A*_b_ values with predicted B and RSA values of the relevant residues (Eq. 3 and 4). We also used Eq. 3 and 4 for de novo prediction of potential B cell epitopes within selected HCMV antigens of known tertiary structures. Specifically, we considered as B cell epitopes those fragments consisting of 9 or more consecutive residues with a *F*_b_ ≥ 1.0 and an *A*_*b*_ ≥ 48%. Peptides fitting these structural criteria are found to be located in highly flexible and solvent-exposed regions of the antigen [25].

### Other procedures

We used BLAST searches [60] against the PDB database subset at NCBI to map B cell epitopes onto 3D-structures and retrieve the relevant PDBs. We also used BLAST searches to determine sequence identity between epitopes and human or human microbiome proteins as described elsewhere [25]. For these searches, we used the NCBI non-redundant (NR) collection of human proteins and the human microbiome protein sequences obtained from the NIH Human Microbiome Project at NCBI (https://www.ncbi.nlm.nih.gov/bioproject/43021). We visualized 3D-structures and produced molecular renderings using the PyMOL Molecular Graphics System, Version 1.2r3pre, Schrödinger, LLC.

## Data Availability

Epitope datasets analyzed in this study were obtained and are available at the IEDB resource (http://www.iedb.org/) and from the corresponding author on reasonable request.

## Abbreviations

Ab: Antibody
gB: Glycoprotein B
gH: Glycoprotein H
gH: Glycoprotein M
gL: Glycoprotein L
HCMV: Human Cytomegalovirus
HLA: Human Leukocyte Antigen
MHC: Major Histocompatibility Complex
pp65: 65 KDa phosphoprotein
RSA: Relative Solvent Accessibility
