# Correction to: Computational assembly of a human Cytomegalovirus vaccine upon experimental epitope legacy

**DOI:** 10.1186/s12859-020-3452-7

**Published:** 2020-03-19

**Authors:** Monica J. Quinzo, Esther M. Lafuente, Pilar Zuluaga, Darren R. Flower, Pedro A. Reche

**Affiliations:** 1 Faculty of Medicine, University Complutense of Madrid, Pza Ramon y Cajal, s/n, 28040 Madrid, Spain; 2 School of Life and Health Sciences, Aston University, Aston Triangle, Birmingham, B4 7ET UK

**Correction to: BMC Bioinformatics**

**https://doi.org/10.1186/s12859-019-3052-6**

After publication of the original article [1], we were notified that legends of Fig. 1 and Fig. 2 have been swapped.

Below the legends are correctly related to the figures.

**Fig. 1:**
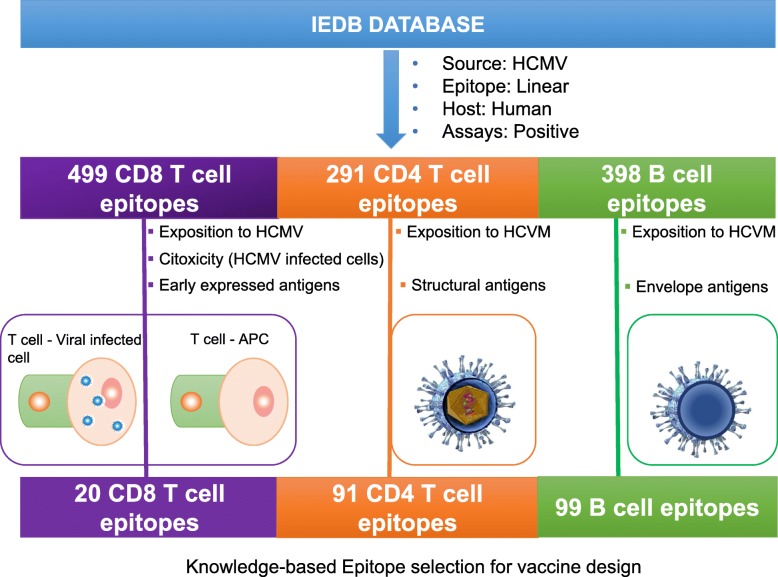
Knowledge-based selection of experimental epitopes for HCMV vaccine design. Experimental epitopes were obtained from IEDB and selected to identify those that are more likely to induce protective immunity in humans. CD8 T cell epitopes were identified upon searches that guarantee that were processed and presented early by APCs (immunogen exposition) and by target cells (mediate cytotoxic activity of cells infected with HCMV). CD4 T cell epitopes were selected for being recognized by HCMV exposed subjects and belonging to structural proteins, so that they will provide early effective help. B cell epitopes were also selected for being recognized by HCMV exposed subjects and mapping onto the ectodomain of envelope proteins so that they can induce neutralizing antibodies

**Fig. 2:**
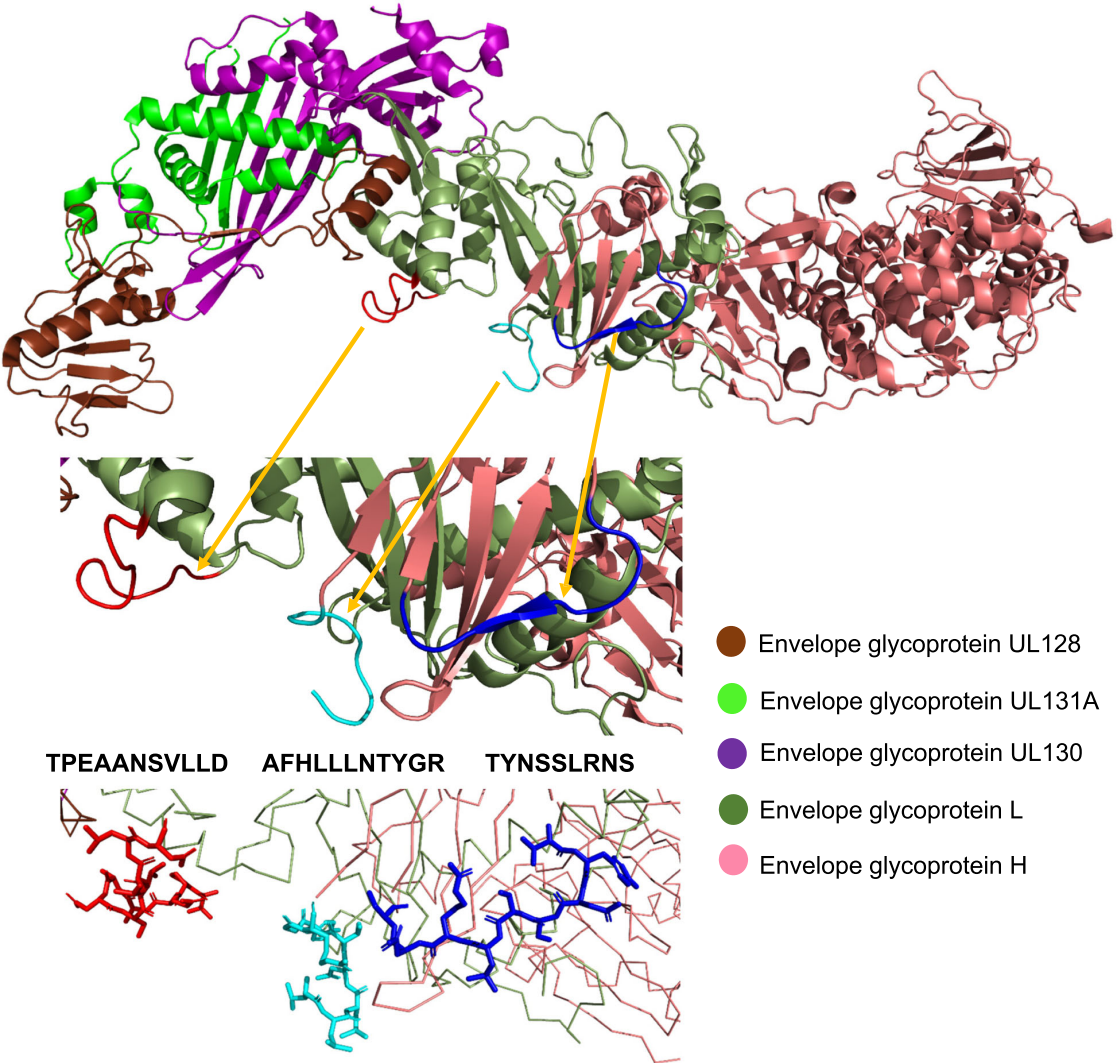
Mapping of predicted (purple and blue) and experimentally defined (red) B cell epitopes on the tertiary structure of the gH and gL as part of the pentameric complex L75/UL115/UL128/UL130/UL131A. B cell epitopes are respresented as sticks over a background of ribbons
